# Nav1.8 and Chronic Pain: From Laboratory Animals to Clinical Patients

**DOI:** 10.3390/biom15050694

**Published:** 2025-05-10

**Authors:** Yu-Feng Xie

**Affiliations:** Neurosciences and Mental Health, The Hospital for Sick Children, Toronto, ON M5G 0A4, Canada; yufeng711@gmail.com

**Keywords:** voltage-gated sodium channel, Nav1.8, dorsal root ganglion, gain-of-function, loss-of-function, chronic pain, excitability, small fiber neuropathy, degeneration

## Abstract

As a subtype of voltage-gated sodium channel and predominantly expressed in the sensory neurons located in the dorsal root ganglion (DRG), the Nav1.8 channel encoded by the *SCN10A* gene is found to have different variants in patients suffering chronic pain or insensitivity to pain due to the gain-of-function or loss-of-function of Nav1.8 channels. In animal models of chronic pain, Nav1.8 is also verified to be involved, suggesting that Nav1.8 may be a potential target for treatment of chronic pain. Another voltage-gated sodium channel, Nav1.7, is also proposed to be a target for chronic pain, supported by clinical findings in patients and laboratory animal models; however, there is no Nav1.7-specific drug that has passed clinical trials, although they demonstrated satisfactory effects in laboratory animals. This discrepancy between clinical and preclinical studies may be related to the differences between humans and laboratory animals or due to the degeneracy in different sodium channels governing the DRG neuronal excitability, which is thought of as the underlying machinery of chronic pain and mostly studied. This review summarizes recent findings of Nav1.8 in chronic pain from clinics and laboratories and discusses the difference, which may be helpful for future investigation of Nav1.8 in chronic pain, considering the dilemma of the Nav1.7 channel in chronic pain.

## 1. Introduction

As an epidemic disease, chronic pain affects up to 40% of people in the world; it is a huge burden to the patients, families, and society, and there is no ideal therapeutic strategy yet [1]. The mechanisms of chronic pain are complex and considered as a result of the enhanced excitability of sensory neurons located in the dorsal root ganglion (DRG) [2]. The excitability of DRG neurons is attributed to multiple voltage-gated sodium channels (VGSCs), including TTX-sensitive VGSCs and TTX-resistant VGSCs [3]. Among these VGSCs, the TTX-sensitive subtype Nav1.7 received more attention than other VGSCs because of clinical findings in patients suffering inherited erythromelalgia, small fiber neuropathy, paroxysmal extreme pain disorder, or congenital insensitivity to pain due to gain-of-function or loss-of-function of Nav1.7 [4]. Therefore, several small molecules targeting Nav1.7 were developed to treat chronic pain and showed satisfactory therapeutic effects in animal models [5]. However, there is no Nav1.7-specific molecule showing satisfactory effect in treating patients with chronic pain in clinic [4,6,7], which encourages researchers to consider another subtype of VGSC, Nav1.8.

NaV1.8, encoded by the *SCN10A* gene, is a subtype of TTX-resistant VGSCs, first identified in DRG and thereby initially termed as a sensory neuron-specific (SNS) channel [8]. Recent studies found that mutation of Nav1.8 is closely associated with chronic pain [9,10,11,12], suggesting Nav1.8 may be a potential target and possible substitution for chronic pain treatment. These results also imply the existence of VGSC degeneracy in DRG neurons in the pathological condition of chronic pain.

Degeneracy is a phenomenon that widely exists in the biological system; it has been found in different types of neurons and plays an important role in neuronal spiking via ion channels [13,14,15]. Our recent study indicates that both Nav1.3, Nav1.7, and Nav1.8, depending on the culturing time, can govern the repetitive spiking in small DRG neurons that respond to nociceptive pain [16]. These results suggest that the excitability of sensory neurons is attributed to a combination of different VGSCs and that degeneracy of ionic channels, including Nav1.8, except for Nav1.7, which has been a focus for chronic pain but does not demonstrate effective therapeutic strategies in clinics [4,6,7,17], should be equally considered when studying chronic pain. Since there are several reviews about Nav1.7 in chronic pain [4,5,6,17], we will focus on the recent findings of Nav1.8 in chronic pain in this review.

## 2. Characteristics of the Nav1.8 Channel

**Figure 1:**
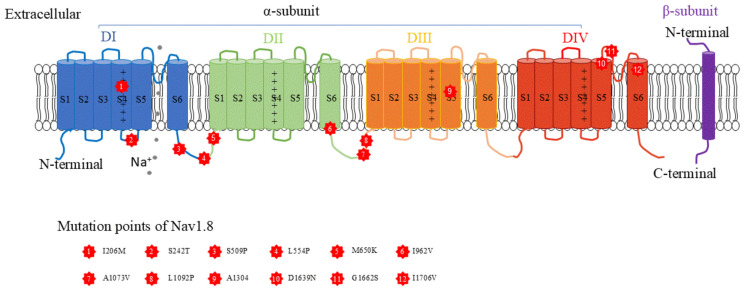
Structure of the Nav1.8 channel and corresponding location of different variants identified in patients with chronic pain.

The Nav1.8 channel is composed of four homologous domains (α-subunits and DI-IV) coded by *SCN10A* and two β-subunits (β1/3 and β2/4) coded by *SCN1-4B*. Each α-subunit contains four voltage-sensitive transmembrane segments (S1-4) and two pre-forming segments (S5-6) (Figure 1). As a member of the Ig family, the β-subunits have an extracellular N-terminal, a transmembrane segment, and an intracellular C-terminal, a total of 1983 amino acids in humans. The β-subunits interact with α-subunits via the N-terminal [18,19]. Recently, the structure of the Nav1.8 channel was investigated by using the cryo-EM technique, and some kinetic features were illustrated. For example, the human Nav1.8 channel demonstrated multiple conformations to change its affinity to the blocker A-803467, which was modulated by the site of Thr397 on S6 and Gly1406 on S6 [20]. Another study identified an unexpected movement of the S4 and S5 helix and found that the S3-S4 linker was the binding site of Nav1.8 blocker protoxin-I [21]. These studies are helpful for designing and developing Nav1.8-specific blockers.

As one of the TTX-resistant VGSCs (Nav1.8 and Nav1.9), Nav1.8 is the first identified SNS channel that is found to be expressed predominantly in small DRG or trigeminal ganglion (TG) neurons [22,23,24]. However, Nav1.8 could not be detected in the superior cervical ganglion (SCG) of SD rats [25]. Using ChR2 floxed to Nav1.8-Cre to label the DRG neurons, it is found that these Nav1.8-ChR2-positive neurons cover Aβ-, Aδ-, and C-fiber mechanoreceptors (low-threshold mechanoreceptors, LTMRs) [26].

The expression of Nav1.8 increases even at E15 and stays stable after postnatal day 7, mainly distributed in TrkA^+^ unmyelinated C-type fibers, myelinated A-type fibers, and a small proportion of IB4^+^ unmyelinated C-fibers [27,28]. These small DRG neurons expressing Nav1.8 also respond to nerve growth factor (NGF) and glial cell-derived neurotrophic factor (GDNF) and express transient receptor potential vanilloid 1 (TRPV1) [28,29]. In addition, the expression of Nav1.8 can be enhanced by the β3 subunit through the interaction between the intracellular C-terminal and the first intracellular loop of the α-subunit, thus transferring the restricted Nav1.8 channel in the endoplasmic reticulum to the cellular surface [30].

Except for expression in peripheral neurons, Nav1.8 is found to be expressed in some central neurons, including neurons located in areas involved in pain. For example, in the limbic circuitry, including the bed nuclei of the stria terminalis, amygdala, and periaqueductal gray, Nav1.8-expressing neurons are identified, and these neurons can respond to noxious stimuli [31]. In addition, Nav1.8 current is also found in pyramidal neurons in the prefrontal cortex [32], and the Nav1.8 translatome in prefrontal neurons can be increased by suppressing transcription factor 4 to regulate neuronal excitability [33]. These studies suggest that Nav1.8 channels are also involved in the central modulation of chronic pain, including the emotional component of pain.

It is proposed that the β subunits bind to different sites of α subunits and modulate the gating of α subunits, therefore affecting the excitability of neurons [34]. In *Xenopus* oocytes expressing human Nav1.8 α-subunit and β1/β3 subunits, it is found that the maximal Nav1.8 current amplitude is down-regulated by the intracellular domain of the β3 subunit, while the inactivation of Nav1.8 current is prolonged by the extracellular domain of the β3 subunit [35]. In addition, the co-expression of Nav1.8 channels can rescue electrogenic properties in SCG neurons due to mutation of Nav1.7 in L858H [25].

## 3. Clinical Findings of Nav1.8 in Chronic Pain

Recently, there were increasing reports of patients suffering chronic pain due to the mutation of the Nav1.8 channel, including gain-of-function and loss-of-function (Figure 1, Table 1), suggesting genomic changes in Nav1.8 may be related to chronic pain.

### 3.1. Gain-of-Function of Nav1.8 Channel

There are several reports about the gain-of-function of Nav1.8 involved in chronic pain. Two women at 24 and 62 years of age carrying the Nav1.8 mutation at G1662S are reported to suffer from idiopathic painful small fiber neuropathy (SFN). The transfection of DRG neurons with G1662S substitution promotes the recovery of Nav1.8 current from inactivation and enhances the neuronal excitability [10], suggesting a gain-of-function of the Nav1.8 channel by G1662S mutation. A 67-year-old man suffering from diabetic neuropathy is identified to carry a mutation of Nav1.8 at S242T, which renders the transfected DRG neurons to be hyperexcitable [37]. In a 43-year-old patient suffering nonpainful peripheral paresthesia of limbs, the pV1287I mutation of Nav1.8 is detected. The mutation of pV1287I results in a right-shift of activation and inactivation, a broader AP, and an increased spiking rate [43], suggesting a mix of gain-of-function and loss-of-function of Nav1.8. These studies indicate that gain-of-function of Nav1.8 is correlated with chronic pain.

### 3.2. Loss-of-Function of Nav1.8 Channel

Similarly, mutations resulting in loss-of-function of Nav1.8 are found to be correlated with chronic pain. Mutation of *SCN10A* in site of D1639N is detected in a 37-year-old woman who suffers severe progressive gastroparesis and chronic pain with SFN. This mutation also induces a significant decrease in the intraepidermal nerve fiber density. Transfection of this mutation significantly decreases the Nav1.8 current but has no effect on the gating characteristics of the Nav1.8 channel, possibly by impairment of trafficking of the Nav1.8 channel to the membrane surface [11,38]. In a 61-year-old man suffering burning and tingling sensations in his legs and feet, mutation I1706V of Nav1.8 is reported. Transfection of small DRG neurons with the I1706V mutant results in hyperpolarization, but a decrease in rheobase and an increase in the spiking rate of neurons [36]. In a 53-year-old female suffering from erythromelalgia, mutation of p.M650K in Nav1.8 is detected. This mutation left-shifts the steady-state fast inactivation of the Nav1.8 channel, decreases the spiking rate of transfected neurons, and broadens the AP duration, but does not significantly change any other gating kinetics of the Nav1.8 channel [12]. Together with the gain-of-function of Nav1.8 in chronic pain, these studies point out a paradox of loss-of-function of Nav1.8 in chronic pain.

### 3.3. Single-Nucleotide Polymorphism of Nav1.8 Channel

In addition, single-nucleotide polymorphism (SNP) is used to screen the association of Nav1.8 with chronic pain. For example, in patients suffering inflammatory bowel disease, the SNP of rs6795970 for *SCN10A* is significantly prevalent in patients with hypoalgesic inflammatory bowel disease, suggesting a link between the genetic mutation of Nav1.8 and visceral pain [42]. Some cohort studies also indicate that the SNP of Nav1.8 is involved in chronic pain. For example, the SNP of rs6795970 is found to be associated with reduced mechanical pain in humans and spiking rate in transfected DRG neurons, suggesting a lose-of-function of Nav1.8 [41]. In a cohort of a retrospective study of patients undergoing sigmoid colectomy, it is found that a polymorphism of Nav1.8 at rs6795970 is associated with lower abdominal pain scores. The rs6795970 mutation also increases the activation threshold of transfected DRG neurons, resulting in loss-of-function of Nav1.8 [39] and correlating with visceral pain of both Crohn’s disease and ulcerative colitis [44]. In another cohort of 309 healthy women, the SNP of rs6801957 shows that the A/A allele carrier has a significantly lower mechanical pain sensation than G/G and G/A allele carriers, suggesting an association between the rs6801957 of Nav1.8 and mechanical pain sensitivity in humans [40]. Furthermore, in a screening of 104 patients suffering from SFN, nine patients are detected to carry seven variants of Nav1.8 (pL554P, Pp939l, Pg940l, pA1056A, pA1304T, pC1523T, and pG1662S), including two variants of A1304T and L554P with gain-of-function [9]. These studies suggest that SNP of Nav1.8 is also involved in chronic pain.

### 3.4. Expression Changes in the Nav1.8 Channel in Patients with Chronic Pain

There are some studies that indicate the involvement of the alteration of Nav1.8 expression in chronic pain. For example, in patients with chronic pain due to myofascial temporomandibular disorders, the expression of Nav1.8 in the masseter muscle-innervating nerves is increased [45]. In patients with chronic pain due to lingual nerve neuromas, the expression of Nav1.8 is significantly higher than in patients without pain [46]. In patients suffering chronic pain due to neuromas, the expression of Nav1.8 in nerves is increased, accompanied by an increase in Nav1.3, Nav1.7, and MAPKs p38 and ERK1/2 [47]. In patients suffering traumatic pain because of brachial plexus injury, the expressions of Nav1.8 and Nav1.9 in the lumbar spine are increased [48]. These studies imply that the increase in the Nav1.8 channel is correlated with chronic pain.

### 3.5. Clinical Trials in Treating Chronic Pain with Nav1.8 Blockers

Considering the close relationship between the mutation of the Nav1.8 channel and chronic pain, Nav1.8 is proposed to be a potential target for the treatment of chronic pain; several small molecules targeting Nav1.8 have been developed and used in preclinical studies, such as LTGO-33 [49,50], A-803467 [51], A-887826 [52], PF-01247324 [53], VX-150 [54], and VX-128 [55]. However, compared to clinical trials of Nav1.7 in chronic pain [6], there are fewer clinical trials testing these blockers targeting Nav1.8 in chronic pain, including only two clinical trials in phase III (Table 2). For example, PF-04531083, a Nav1.8-specific drug constructed by Pfizer, is used to treat post-surgical dental pain with a single dose in phase II and is terminated due to futility based on results of internal analysis (NCT01512160).

VX-150 is a drug developed by Vertex Pharmaceuticals to treat chronic pain by targeting Nav1.8 through oral or intravenous administration. In a phase II clinical trial, long-term administration of VX-150 at 1250 mg per day for 6 weeks significantly attenuated the pain due to SFN (NCT03304522). Another phase II clinical trial tested the effect of VX-150 on osteoarthritis pain (NCT02660424); however, there is no posted result. In 2023, Vertex announced that another Nav1.8-specific drug, VX-548 (also known as Suzetrigine), showed a significant reduction in chronic pain in a phase II study of patients suffering diabetic peripheral neuropathy after 12 weeks of oral administration of VX-548 at three different doses when compared with placebo (NCT05660538), and three clinical trials at phase III are recruiting patients with diabetic peripheral neuropathy. However, there is no accessible public publication about the drug in treating chronic pain, and VX-548 (Suzetrigine) was approved only for acute pain by the FDA in 2025 [56,57].

In a phase III clinical study with patients suffering from SFN (NCT01911975) [58], the effects of lacosamide, which targets Nav1.3, Nav1.7, and Nav1.8 [59], are studied in patients with SFN, and the results indicate that lacosamide could significantly decrease the average pain score and improve sleep and surface pain intensity, suggesting a complex involvement of Navs in chronic pain.

Lidocaine also attenuates the chronic pain in a patient suffering SFN due to the mutation of Nav1.8 D1639N [11]. In a case series study, topical application of the Nav1.8 blocker ambroxol effectively reduced spontaneous pain, movement-related pain, allodynia, and hyperalgesia in eight patients with complex regional pain syndrome [60].

### 3.6. Study of Chronic Pain with iPSCs from Patients

The technique of human-induced pluripotent stem cells (iPSCs) has been used in multiple diseases since it was developed a decade ago [61]. This strategy can provide a “disease in a dish” model for studies of different diseases because these iPSCs contain the genetic background and native transcriptional machinery of the donors. This feature of iPSCs thus allows study of the machinery of diseases without the artifacts arising from overexpression systems or transgenic animals. Using iPSC-derived sensory neurons from patients with congenital insensitivity to pain, McDermott et al. [62] found that Nav1.7-specific blocker PF-05089711 could not change the excitability of neurons derived from iPSC sensory neurons, while another blocker, BIIB704, could decrease the excitability of iPSC-derived sensory neurons, suggesting a challenging translation of VGSCs in pain therapeutics [63]. In addition, it is found that virtual Nav1.8 and Nav1.9 by dynamic clamp contribute to the repetitive spiking in iPSC-derived sensory neurons, and a small change in Nav1.8 gating biophysics equivalent to the gain-of-function of Nav1.8 mutation in I1706V significantly changes the excitability [64]. Recently, iPSCs from patients suffering from SFN and carrying the T365N mutation of *SCN10A* were successfully programed, providing a hopeful application and potential tool to investigate the underlying mechanisms of SFN by Nav1.8 mutation [65]. These results provide encouraging potential in studying chronic pain with iPSC-derived neurons from Nav1.8 mutant patients.

## 4. Preclinical Study of Nav1.8 in Chronic Pain

Preclinical studies in animals indicate that Nav1.8 is involved in chronic pain at different dimensions.

### 4.1. Electrophysiological Studies of Nav1.8 in Animals with Chronic Pain

Mutation of Scn10a^Psm/Psm^ results in enhancement of Nav1.8 current and AP and hyperexcitability of small DRG neurons [66,67]. In the DRG neurons carrying Nav1.8 mutations at G1662S and T790A, the TTX-R current is increased by impairing the inactivation, resulting in enhancement of excitability with lower rheobase and higher spiking rate [68]. In Nav1.8 knockout mice, the response of spinal dorsal horn neurons to mechanical stimuli is absent, but not to thermal stimuli [69]. Inflammation in the lumen of the distal colon can increase the Nav1.8 currents in small DRG neurons [70]. Chronic constriction injury (CCI) right-shifts action of Nav1.8 current by 5.3 mV and left-shifts inactivation of Nav1.8 current by 10 mV, rendering DRG neuron hyperexcitability [71]. In small DRG neurons from mice with peripheral neuropathy induced by vincristine, the excitability is increased, accompanied by an increase in Nav1.8 current and hyperpolarization of the action of the Nav1.8 channel [72].

In the inflammatory pain model induced by complete Freund’s adjuvant (CFA), the action of Nav1.8 in DRG neurons is shifted to hyperpolarization, meaning decreased current threshold and increased excitability [73]. Nav1.8-specific blocker PF-01247324 inhibits Nav1.8 currents in human DRG neurons and small-diameter rodent DRG neurons in a frequency- and state-dependent manner and reduces the excitability of DRG neurons of both rats and humans [53].

### 4.2. Transcriptional and Translational Changes in Nav1.8 in Animals with Chronic Pain

The change in Nav1.8 expression has been confirmed at both protein and mRNA levels in different chronic pain models. For example, the expression of Nav1.8 in rats showing neuropathic pain induced by chronic compression of DRG is significantly increased [74]. In inflammatory pain induced by CFA and neuropathic pain induced by sciatic nerve injury (SNI) or chronic compression of DRG, the expressions of Nav1.8 mRNA and protein are significantly increased [73,74,75]. In the neuropathic pain model of rats induced by paclitaxel, which is widely used for the treatment of different cancers, the expression of Nav1.8 protein is significantly increased [76]. In cancer pain induced by experimental tumor, the expression of Nav1.8 protein is significantly increased in DRG neurons [77].

However, a series of studies from the Waxman group and other groups show that the Nav1.8 mRNA is significantly decreased in DRG neurons after axotomy in rats [78,79] and that Nav1.8 mRNA can be downregulated by axotomy or SNI, which can be restored by NGF and GDNF [70,80]. Other studies also indicate a decrease in Nav1.8 mRNA and protein in DRG of rats with pain induced by CFA, nerve ligation, or chronic constriction injury [8,71]. Furthermore, other studies indicated that Nav1.8/SNS mRNA is only slightly changed by NGF or CFA but is significantly decreased by peripheral, not central, DRG axotomy or streptozotocin-induced neuropathy [8,81]. This discrepancy may be due to different species and/or methodology and suggests that the transcriptional and translational changes may not be necessarily correlated under pathological conditions [82], which is consistent with our study indicating that Nav1.8 mRNA does not significantly correlate with the function of the Nav1.8 channel in small DRG neurons [16].

### 4.3. Behavioral Studies of Nav1.8 in Animals with Chronic Pain

Behavioral studies in transgenic animals indicate that Nav1.8 is involved in chronic pain. In mutant mice carrying the gain-of-function of SCN10A variant G1663S, the mice demonstrated higher sensitivity to mechanical and thermal stimulations [83]. In mutant mice carrying Scn10a^Psm/Psm^, a gain-of-function of Nav1.8, noxious chemical and mechanical stimuli induce hyperalgesia [66] and cold pain [67] more than in control mice. Similarly, inhibition of DRG neurons by chemogenetics or specific antisense oligodeoxynucleotides targeting Nav1.8 attenuates the hyperalgesia in experimental osteoarthritis induced by destabilization of the medial meniscus [84,85,86] and attenuates mechanical allodynia in inflammatory pain induced by capsaicin and zymosan and neuropathic pain induced by SNI [87]. In Nav1.8 knockout mice, the response to mechanical stimuli but not to thermal stimuli is absent [69]. In contrast, some studies indicate that silencing Nav1.8-expressing neurons could aggravate chronic pain. For example, in FLExPSAM-GlyR mice, inactivation of Nav1.8-positive nociceptors by chemogenetic silencing can block the mechanical and thermal pain induced by CCI or cancer [88]. In another study, suppression of Nav1.8 by overexpression of miRNA 3584-5p aggravates mechanical and thermal allodynia induced by CCI [89].

Pharmacological studies also support the role of Nav1.8 in different chronic pain models by using different Nav1.8-specific blockers. Blockade of Nav1.8 with the Nav1.8-specific blocker A-803467 attenuates the mechanical hyperalgesia of joints induced by lysophosphatidic acid [90], mechanical/cold allodynia, and spontaneous pain in both young (6 months) and old (24 months) rats induced by CFA [91]. Another Nav1.8-specific blocker, PF-01247324, significantly attenuates the thermal hyperalgesia induced by carrageenan, mechanical hyperalgesia induced by CFA, and mechanical allodynia induced by spinal nerve ligation (SNL) [53]. Nav1.8-specific blocker A-887826 can significantly attenuate the allodynia induced by SNL in rats [52]. Inhibition of Nav1.8 by dexpramipexole or plerixafor attenuates neuropathic pain induced by chemotherapy drugs oxaliplatin and streptozotocin, chronic constriction of the sciatic nerve in mice [92], or chronic compression of DRG [74].

### 4.4. Studies on Mutations in Nav1.8 in Animal Models

The transgenic mice carrying the mutation of Nav1.8 in G1662S show hypersensitivity to noxious stimuli, and the DRG neurons show hyperexcitability, consistent with clinical findings that this mutant of Nav1.8 is correlated with painful SFN [83]. In another mouse strain of Possum, the Nav1.8 mutant T790A renders mice to show hyperalgesia with mechanical and cold stimuli and DRG neurons to hyperexcitability [67,68]. In addition, the DRG neurons from mutants of *SCN10A*^Psm/Psm^ show hyperexcitability to mechanical stimuli, and the mutant mice show hyperalgesic responses to frankly noxious mechanical stimuli and noxious chemical stimuli with mustard oil [66].

Global knockout of *SCN10A* in mice does not change the response to noxious heat in burn models or CFA models, to inflammatory pain models by acetic acid, formalin, or carrageenan, or to neuropathic pain by SNI or CCI [93,94], when compared to littermates. However, deletion of Nav1.8 does change the response of mice to extreme low but not mild cold stimuli [95] and blocks the mechanical hyperalgesia induced by NGF but not PGE2 or SNI [96]. In visceral pain, deletion of Nav1.8 attenuates painful response to intracolonic application of mustard oil or capsaicin, but not to acetylcholine or cyclophosphamide cystitis [97]. In combined deletion of both Nav1.7 and Nav1.8, the animals develop normal neuropathic pain but not inflammatory pain [98]. These studies indicate a complex of Navs in chronic pain.

## 5. Prospective Study of Nav1.8 in Chronic Pain

### 5.1. Differences Between Human and Animal Studies

Although the involvement of Nav1.8 in chronic pain is supported by clinical findings and preclinical studies in rodents, there is no Nav1.8-targeting drug demonstrating effective therapy for chronic pain. The gap between clinic findings in humans and laboratory results from animals makes us rethink the problem, although Nav1.8 shows a high homologous relationship between humans and rodents [24]. For example, a cross-species study of DRG and TG cells demonstrates similar transcriptomic profiles of sensory neuron subtypes across vertebrates but notable differences in the expression of functionally important neuropeptides and channels [99]. A series of studies from the Waxman group show that Nav1.8 mRNA is significantly decreased in DRG neurons after axotomy in rats [78,79], but Nav1.8 protein is significantly increased in neuromas from patients suffering chronic neuropathic pain due to amputation/fracture [47]. In a comparative study of healthy humans and mice, the DRG neurons show different expressions of VGSCs, i.e., higher Nav1.7 (50%) and lower Nav1.8 (12%) in humans, and higher Nav1.8 (45%) and lower Nav1.7 (18%) in mice [100]. Another study also confirms the difference in DRG neurons between humans and mice, i.e., the ratio of TrkA^+^ DRG neurons expressing Nav1.8 is 50.6 ± 2.0% in mice and 69.8 ± 2.0% in humans [101]. Additionally, although the Nav1.8 channel demonstrates a similar activation curve in humans and rats, the inactivation curve of the Na1.8 channel in humans shows more voltage dependence in hyperpolarization and easier inactivation. The Nav1.8-specific blocker A-803467 also displays higher affinity under the inactivated state for the human Nav1.8 channel than the rat Nav1.8 channel [30]. Recognizing these differences between humans and animals may be helpful in developing novel pain therapeutics targeting Nav1.8.

Due to the sequence differences between humans and rodents, it is possible that Nav1.8 blockers may produce different results between clinical and preclinical studies. Recently, a rodent model with expression of the human Nav1.8 channel in rats was used to screen molecules that target chronic pain, including inflammatory pain (CFA) and neuropathic pain (SNI), and it also identified MSD199 as a Nav1.8 inhibitor with potential analgesic effects [102]. In addition, using non-human primates to investigate the effects of Nav1.8 blocker MSD199 on chronic pain has been performed and demonstrated promising analgesic effects [103]. These strategies may facilitate the study of the Nav1.8 channel in treating chronic pain.

### 5.2. Degeneracy

Degeneracy is the ability to achieve the same outcomes by different elements [104] and exists in different biological systems such as pain [105,106]. Consistently, our study indicates that the excitability of DRG nociceptor neurons can be equivalently determined by Nav1.3, Nav1.7, and Nav1.8, depending on the culture time [16]. In addition, another TTX-R Nav1.9, which is also predominantly expressed in DRG, is also implicated in chronic pain [107,108] or no pain [109,110] in patients and in animal models [46,111,112,113]. These studies suggest that targeting one type of Nav channel may be insufficient for treating chronic pain. Consistently, in a chronic inflammatory pain model induced by CFA in the knee, Nav1.7, Nav1.8, and Nav1.9 are increased at different time courses [114]. Considering the failure of Nav1.7-targeting drugs in clinical trials despite the success in preclinical studies [4,6,17], multiple-drug targeting [106,115] may be considered when studying chronic pain. This proposal is supported by a clinical trial that targeting Nav1.3, Nav1.7, and Nav1.8 by lacosamide attenuates pain in SFN (NCT01911975) [58].

### 5.3. Machine Learning in Developing Nav1.8-Targeting Drugs in Chronic Pain

Computational science and machine learning are powerful tools that have been recently applied in the study of pain and Nav channels. For example, by using systematic bioinformatics to analyze the neuropathic pain datasets (GSE24982 and GSE63442) from the Gene Expression Omnibus database, the Nav1.8 coding gene *SCN10A* was identified as a biomarker for neuropathic pain [116]. Computational modeling could also be used to study the biophysical spatial extent of axons, ion channels in axons, and spontaneous spiking related to chronic pain [117]; the binding properities of analgesics under different conformational states [118]; Nav1.8 kinetics [119]; the influence of the densities of Nav1.7/1.8 in axons on the conduction of mechano-sensitive C-nociceptors and mechano-insensitive C-nociceptors related to pain; and the contribution of the NGF-induced change in nociceptor phenotypes [120].

Computational models are also used to investigate the interaction between Nav1.8 and Nav1.7. By using machine learning algorithms with protein–protein interaction (PPI) and drug–target interaction (DTI) networks, screening potential therapeutic compounds for chronic pain can be promoted for their properties of absorption, distribution, metabolism, excretion, and toxicity [121]. It is also found, using computer simulation, that coexistence of Nav1.7 and Nav1.8 increases the AP amplitude subthreshold and repetitive firing, while upregulation of Nav1.8 could attenuate the Nav1.7 current [122], further supporting the degeneracy hypothesis of different Navs in chronic pain.

## 6. Conclusions

In summary, following the advances of Nav1.8 blockers in clinical acute pain studies (NCT05034952, NCT04977336, NCT03764072, NCT03206749, NCT06628908, and NCT06696443) [54,55,123], application of iPSCs to patients suffering chronic pain and rodents expressing human Nav1.8 (see above), and computational modeling in the sensory system [119,124], the application of Nav1.8 blockers in chronic pain will increase in the future.

## Figures and Tables

**Table 1:** The summary of Nav1.8 genetic mutation in humans.

References	PMID	Age (yrs)	Sex	Mutation Focus	Function	Clinical	E-Phys
[11]	30099632	32/37	F/F	D1639N	Loss	SFN	
[10]	24006052	24/62	F	G1662S	Gain	SFN	
[36]	23986244	61	M	I1706V	Loss	SFN	Transfected DRG neuron, 40–55 h, CsCL-based, small
[9]	23115331	67, 39, 69	2 M/1 F	L554P, A1304T	Gain	iSFN	
[37]	30135145	67	M	S242T	Gain	SFN	Transfected DRG NGF/GDNF, 40–48 h
[38]	26711856	37	F	D1639N	na	SFN: severe progressive gastroparesis and diffuse painful small fiber sensory neuropathy	
[39]	31642403	166	M/F	A1073V		Lower abdominal pain scores	Male SD rat, SCG no trophic factors, 16–24 h, NMG-based, small
[40]	29448912			rs6801957-G/A		decreased experimental mechanical pain sensitivity	
[41]	27590072	22.6	187 M/309 F	A1073V	Loss	Higher thresholds for mechanical pain	Shifts channel activation by −4.3 mV and accelerates inactivation, reduces repetitive firing of DRG neurons, and lowers mechanical pain sensitivity
[42]	30538988	41.7	58 M/63 F	A1073V	Loss	Hypoalgesic IBD patients	
[12]	27598514	53	M	p.M650K	Loss	Erythromelalgia: increased activity-dependent slowing in CMi and less spontaneous firing in peripheral nerve fibers than non-mutant erythromelalgia	P3-6d Wistar rat, f/m, small DRG neurons, culture 1 day. Shifted steady-state fast inactivation of Nav1.8 to hyperpolarization, increased AP duration, and reduced AP rate

**Table 2:** Clinical trials in treating chronic pain with Nav1.8-specific blockers.

NCT	Drug	Clinical Trial	Pain Model	Gender	Age	Sample Size	Dose	Routine	Status
015121608	PF-04531083	Phase II	Post-surgical dental pain	M	18–55	90	1–2 g, single	Oral	Terminated for futility based on results of internal analysis
03304522	VX-150	Phase II	SFN	M/F	18–80	89	1.25 g, daily, 6 wks	Oral	
06176196	VX-548	Phase II	Painful lumbosacral radiculopathy	M/F	18–70	Estimate 200	12 wks	Oral	Recruiting
05660538	VX-548	Phase II	Diabetic peripheral neuropathy	M/F	18–80	192	23, 46, 69 mg, qd, 12 wks	Oral	Significantly reduced pain
06628908, 06696443	Suzetrigine	Phase III	Diabetic peripheral neuropathy						Recruiting

## Abbreviations

CCI, chronic constriction injury; CFA, complete Freund’s adjuvant; DRG, dorsal root ganglion; DTI, drug–target interaction; GDNF, glial cell-derived neurotrophic factor; iPSCs, induced pluripotent stem cells; LTMR, low-threshold mechanoreceptor; NGF, nerve growth factor; PPI, protein–protein interaction; SFN, small fiber neuropathy; SNI, sciatic nerve injury; SNL, spinal nerve ligation; SNP, single-nucleotide polymorphism; SNS, sensory neuron-specific; TG, trigeminal ganglion; SCG, superior cervical ganglion; VGSC, voltage-gated sodium channel; TRPV1, transient receptor potential vanilloid 1.
